# Factors influencing quality of life in children with atopic dermatitis and their caregivers: a cross-sectional study

**DOI:** 10.1038/s41598-019-51129-5

**Published:** 2019-11-05

**Authors:** Xiaomeng Xu, Louise Sandra van Galen, Mark Jean Aan Koh, Ram Bajpai, Steven Thng, Yik Weng Yew, Valerie Pui Yoong Ho, Uma Alagappan, Krister Sven Ake Järbrink, Josip Car

**Affiliations:** 1 Centre for Population Health Sciences (CePHaS), Lee Kong Chian School of Medicine, Nanyang Technological University, Singapore, Singapore; 2 Section of Acute Medicine, Department of Internal Medicine, Amsterdam UMC location VUmc, Amsterdam, The Netherlands; 3 Dermatology Service, KK Women’s and Children’s Hospital, Singapore, Singapore; 4 National Skin Centre, Singapore, Singapore; 5 Skin Research Institute of Singapore, Singapore, Singapore

**Keywords:** Skin diseases, Quality of life, Paediatric research

## Abstract

Better understanding of atopic dermatitis’ effect on quality of life could enhance current management and therapeutic strategies. Studies investigating factors related to the health-related quality of life (HRQOL) of children with atopic dermatitis and their caregivers are limited. This cross-sectional study included 559 children (<16 years) with atopic dermatitis and their caregivers. Disease severity was associated with infants’ HRQOL (moderate: IRR: 1.42, 95% CI 1.20–1.67; severe: IRR: 1.72, 95% CI 1.32–2.24). Age and disease severity were associated with children’s HRQOL (age: IRR: 0.99, 95% CI 0.98–1.00; moderate: IRR: 1.08, 95% CI 1.02–1.14). Quality of life subdomains itching/scratching, emotional distress and sleep disturbance were most reported and increased with higher disease severity. Both caregivers’ mental and physical health were negatively affected by children’s HRQOL (physical: IRR: 0.99, 95% CI 0.99–1.00; mental: IRR: 0.98, 95% CI 0.97–0.99). Sociodemographic characteristics (gender, ethnicity, educational attainment of carers, number of children) did not demonstrate significance in children’s HRQOL model. In conclusion, current atopic dermatitis diagnostics and treatment have to be extended to the factors influencing both children’ as their caregivers’ quality of life and adapting management accordingly. Itching/scratching, emotional distress and sleep disturbance deserve attention. Sociodemographic characteristics in children’s HRQOL models also merit attention in further research.

## Introduction

Atopic dermatitis, also called eczema, is one of the most common chronic inflammatory skin conditions in children^1,2^. The main problems of atopic dermatitis include intractable itching and changed appearances^3^. The treatment regimen, including the frequent application of topical creams, can be complex, uncomfortable and stressful for children and their caregivers^4^. Several studies have reported how atopic dermatitis burdens the quality of life of children and their caregivers^5,6^. Children with atopic dermatitis might suffer from sleep loss, irritability, anxiety, lowered self-esteem, and psychological impairment^7^. As for their caregivers, studies have identified significantly higher levels of anxiety and depression due to disruption of sleep and absence from work^8^.

The prevalence of atopic dermatitis in Singaporean school children aged 7 to 12 years is 20.8%^9^, which is relatively high compared to other developed countries (US: 13%; Europe: 15%)^10–13^. A community-based study among Singaporeans demonstrated a high prevalence of atopic dermatitis as well as a low quality of life in adults and children compared with other skin conditions^13^. A more recent study showed that Asian adolescents suffer significant psychosocial impact from this skin condition^12^. It addressed individual domains of quality of life but further analysis of correlated social and clinical factors was not performed due to small sample size^12^. Although clinicians are intuitively concerned whether more severe atopic dermatitis in a lower socioeconomic class family may lead to poorer quality of life, only one small sample study has so far explored it^14^.

Better understanding of atopic dermatitis’ effect on quality of life and the contributing factors can enhance current management and therapeutic strategies. Given the fact that one in five children are affected globally^11^, more empirical in-depth research needs to be conducted to examine factors influencing quality of life. To address this gap, the purpose of this study is to investigate the impact of atopic dermatitis on HRQOL in a high prevalence area and to assess its related factors.

## Results

### Patient characteristics

**Figure 1:**
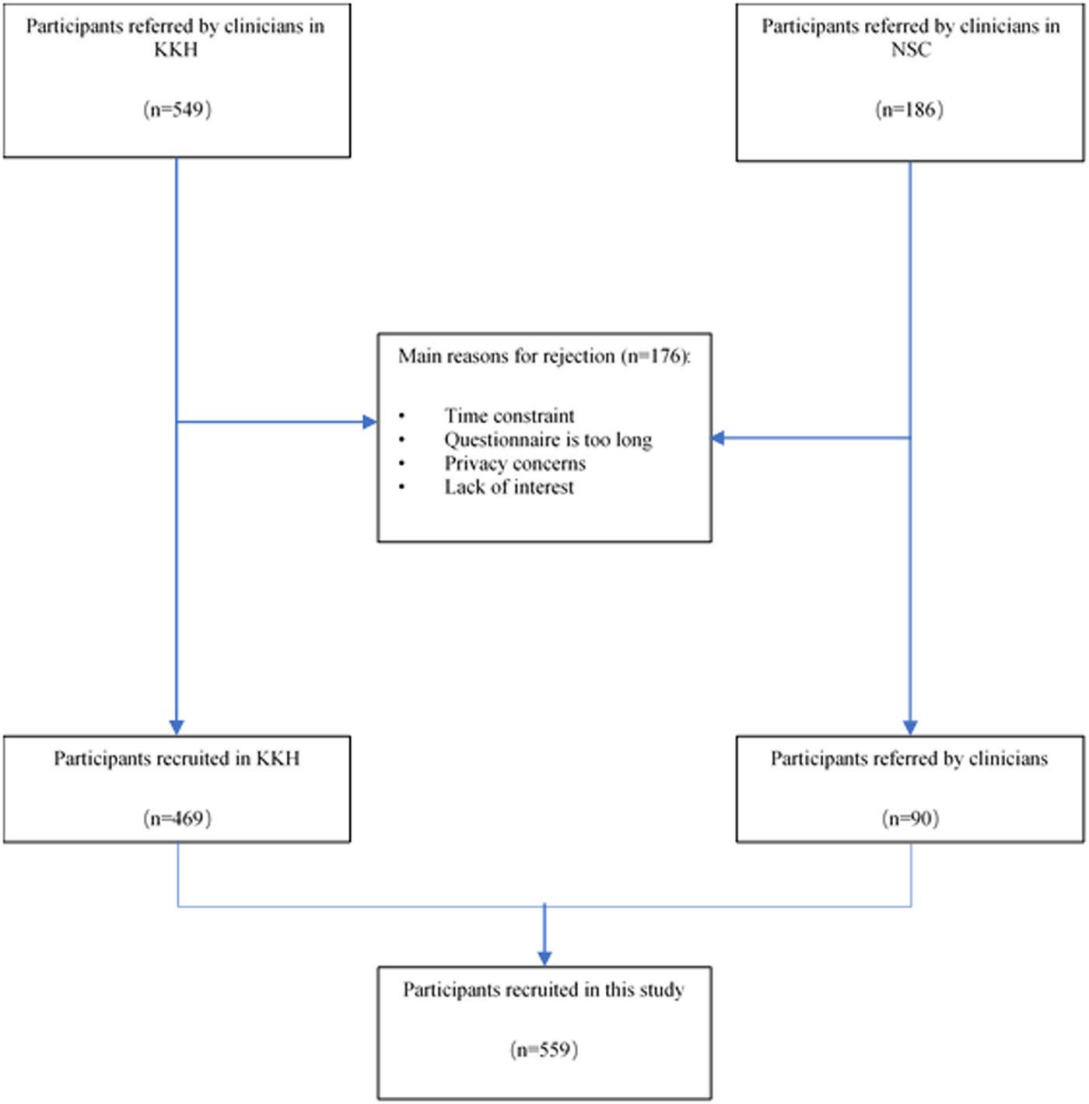
Patient recruitment workflow.

The characteristics of the participants are summarised in Table 1. In total, of 735 eligible children, 559 patients and their caregivers participated (76%). Reasons for exclusion are listed in Fig. 1. The study sample consist of 250 infants (age 0–4 years old) and 309 children (5–16 years old) (50% boys and 50% girls), with a mean age (±SD) of 6.6 ± 4.6 years old. Disease severity was mild in 56% of the cases, moderate in 24% and severe in 11% (in 46 cases, disease severity could not be retrieved). Seventy-two percent of the participants were Chinese, Indian and Malay participants accounted for 16% and 6% respectively. A great majority of caregivers (81%) were educated to at least tertiary-level educational attainments. The majority were employed (81%), 19% were unemployed or retired.

**Table 1:** Background characteristics of AD patients and their caregivers.

Characteristics	Total (n = 559)	Gender		P-value
		Male (n = 282)	Female (n = 277)	
**Child’s age (years) (Mean ± SD)**	6.61 ± 4.55	6.91 ± 4.72	6.30 ± 4.36	0.11
**Ethnicity, n (%)**				0.42
Chinese	404 (72.3)	195 (69.2)	209 (75.5)	
Indian	89 (15.9)	50 (17.7)	39 (14.1)	
Malay	33 (5.9)	19 (6.7)	14 (19.1)	
Others	33 (5.9)	18 (6.4)	15 (5.4)	
**Duration of disease (years) (Mean ± SD**)	3.51 ± 3.62	3.75 ± 3.82	3.27 ± 3.40	0.12
Disease severity*, n (%)				0.45
Mild	316 (56.5)	161(57.1)	155 (56.0)	
Moderate	133 (23.8)	61 (21.6)	72 (26.0)	
Severe	64 (11.5)	35 (12.4)	29 (10.8)	
**Caregiver’s highest achieved educational attainment, n (%)**				0.55
Primary and secondary	103 (18.4)	57 (20.2)	46 (16.6)	
Polytechnic and professional	181 (32.4)	89 (31.6)	92 (33.2)	
University and above	275 (49.2)	136 (48.2)	139 (50.2)	
**Caregiver’s employment status, n (%)**				0.72
Employed	452(80.9)	231 (81.9)	221 (79.8)	
Unemployed/retired	107(19.1)	51 (18.1)	56 (20.2)	
Type of housing*, n (%)				0.29
Public housing 1–3 rooms	68 (12.2)	36 (12.8)	32 (11.6)	
Public housing 4–5 rooms	361 (64.8)	189 (67.0)	172 (62.1)	
Private housing (condo, landed property)	128 (23.0)	57 (20.2)	71 (25.6)	
**Number of children in the family, n (%)**				0.05
1	175 (31.3)	97 (34.4)	78 (28.2)	
2	231 (41.3)	120 (42.6)	111 (40.1)	
> = 3	153 (27.4)	65 (23.1)	88 (31.8)	
**Smoking in family, n (%)**				0.90
Yes	94 (16.8)	48 (17.0)	46 (16.6)	
No	465 (83.2)	234 (83.0)	231 (83.4)	

^*^Missing data: Disease severity, n = 46; Type of housing, n = 2.

### Children’s and their caregivers’ quality of life

**Figure 2:**
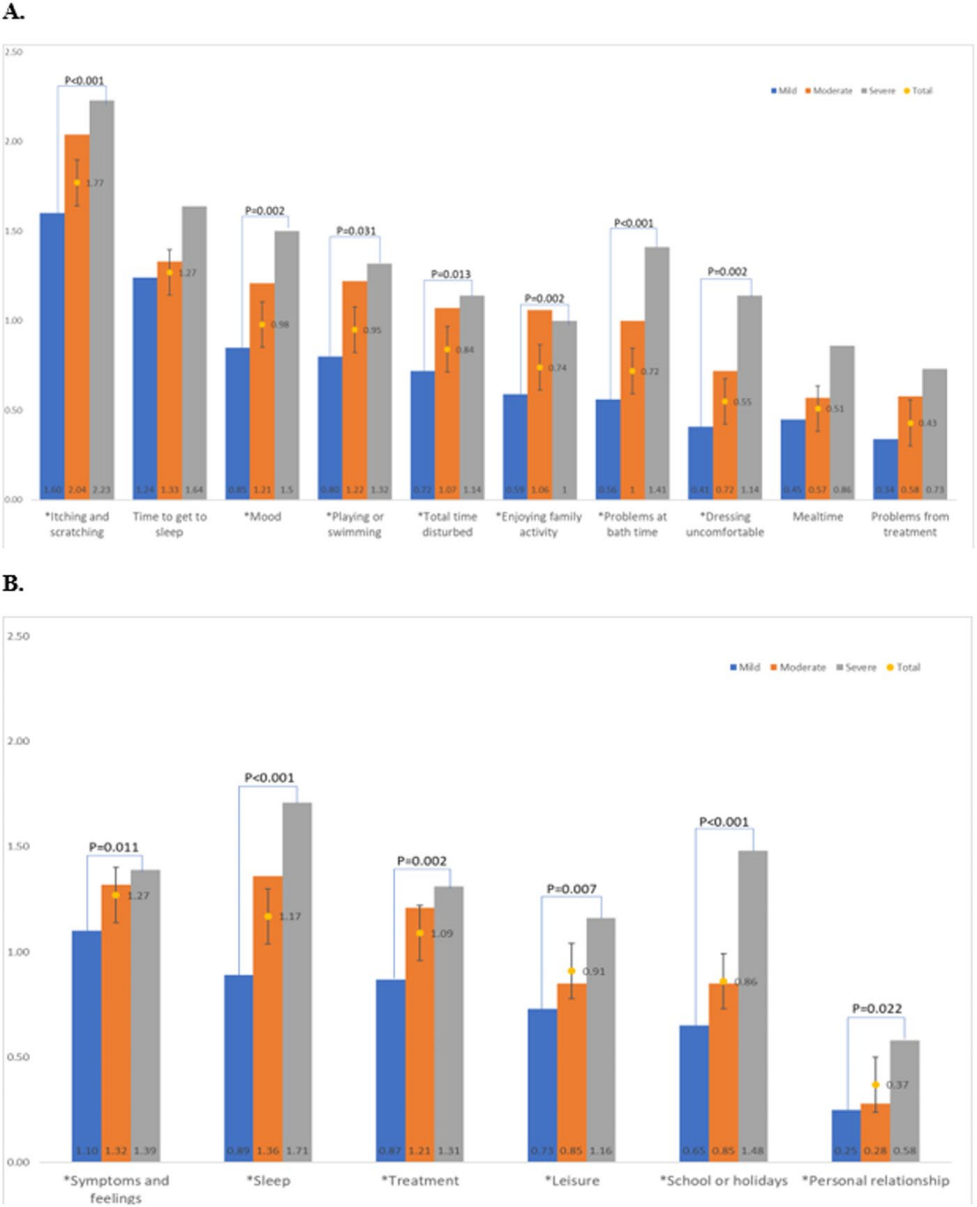
Association between mean score of IDQOL and CDLQI reported subdomains and children’s disease severity. (**A**) IDQOL subdomains (X-axis: subdomains; Y-axis: mean score of IDQOL). (**B**) CDLQI subdomains (X-axis: subdomains; Y-axis: mean score of CDLQI). *Significant differences in quality of life between different severity groups (missing data: n = 46). IDQOL: The score ranges from 0 to 30. A higher score indicates a greater degree of quality of life impairment. CDLQI: The score ranges from 0 to 30. A higher score indicates a greater degree of quality of life impairment.

Mean scores of overall HRQOL were 8.76 ± 5.76 and 8.76 ± 5.46 for IDQOL and CDLQI respectively. Two quality of life subscales with major impact on infants were itching and scratching (mean ± SD: 1.77 ± 0.84) and sleep disturbances (mean ± SD: 1.27 ± 0.82) (Fig. 2). For CDLQI scales, symptoms and sleep disturbance were also the subscales with the largest impact (symptoms: mean ± SD: 1.22 ± 0.68; sleep disturbance: mean ± SD: 1.17 ± 1.07) (Fig. 2). Significant differences in quality of life between different severity groups were observed among infants and children. Figure 2 shows that several IDQOL subdomain scores were increasingly impaired when disease severity increased (itching and scratching (P < 0.001), mood (P = 0.002), total time disturbed (P = 0.013), playing or swimming (P = 0.031), enjoying a family activity (P = 0.002), dressing uncomfortable (discomfort with clothing) (P = 0.002) and problems at bath time (P < 0.001)). CDLQI subscale domains were also increasingly impaired when the severity of the condition increased (symptoms and feelings (P = 0.011), leisure (P = 0.007), school or holidays disturbance (P < 0.001), personal relationship (P = 0.022), sleep affected (P = 0.002), and treatment (P < 0.001)).

Scores in the mental and physical health domains of the RAND-36 for the 559 caregivers were 38.52 ± 14.27 and 50.48 ± 9.69 respectively. Compared to RAND-36 scores in previous studies exploring the relationship between childhood disease and its impact on caregivers/parents, the data indicated that the mental health scores of caregivers looking after children with atopic dermatitis were poorer than those of caregivers of children with psychiatric disorders and equivalent to mothers looking after children with severe developmental disability and physical problems such as heart disease^8,15,16^.

### Factors related to children’s quality of life

A regression model was constructed to assess the relationship between sociodemographic variables and the HRQOL in children (Table 2). The model suggested that moderate and severe atopic dermatitis significantly decrease quality of life in IDQOL (moderate: IRR: 1.42, 95% CI: [1.20, 1.67], P < 0.001; severe: IRR: 1.72, 95% CI: [1.32, 2.24], P < 0.001). In CDLQI, moderate atopic dermatitis significantly decreases quality of life while no similar result was observed in severe cases (IRR: 1.08, 95% CI: [1.02, 1.14], P = 0.01). Children’s age was found to be associated with their quality of life with older children having lower quality of life (IRR: 0.99, 95% CI: [0.98, 1.00], P = 0.02). The remaining sociodemographic characteristics (ethnicity, educational attainment, number of children in the family and type of housing) did not demonstrate significance in the children’s HRQOL model.

**Table 2:** Multivariable regression analysis with factors associated with the children and caregivers’ quality of life.

Variables	Children HRQOL						Caregivers RAND 36					
	IDQOL			CDLQI			Physical health			Mental health		
	IRR	95% CI	P-value	IRR	95% CI	P-value	IRR	95% CI	P-value	IRR	95% CI	P-value
Age of child (years)	1.02	[0.98, 1.06]	0.44	0.99	[0.98, 1.00]	0.02	1.01	[1.00, 1.01]	0.03	1.00	[0.99, 1.01]	0.44
**Gender**												
Male	1(Ref)											
Female	1.09	[0.93, 1.27]	0.31	0.99	[0.94, 1.05]	0.80	0.99	[0.96, 1.03]	0.69	1.00	[0.94, 1.06]	0.94
**Disease duration (years)**	1.01	[0.98, 1.05]	0.51	1.00	[1.00, 1.01]	0.16	1.00	[0.99, 1.00]	0.44	1.00	[0.99, 1.01]	0.60
Disease severity*												
Mild	1(Ref)											
Moderate	1.42	[1.20, 1.67]	p < 0.001	1.08	[1.02, 1.14]	0.01	0.98	[0.94, 1.02]	0.35	0.97	[0.90, 1.05]	0.49
Severe	1.72	[1.32, 2.24]	p < 0.001	1.05	[0.96, 1.15]	0.29	0.94	[0.89, 0.99]	0.03	1.03	[0.93, 1.15]	0.57
**Ethnicity**												
Chinese	1 (Ref)											
Indian	1.12	[0.91, 1.38]	0.30	1.02	[0.95, 1.11]	0.56	0.93	[0.88, 0.97]	0.003	1.00	[0.90, 1.10]	0.97
Malay	1.28	[0.91, 1.80]	0.15	1.01	[0.94, 1.08]	0.82	0.95	[0.88, 1.02]	0.13	1.03	[0.89, 1.20]	0.65
Others	1.28	[0.90, 1.81]	0.17	1.03	[0.93, 1.14]	0.58	1.03	[0.96, 1.11]	0.41	1.04	[0.88, 1.23]	0.67
**Educational attainment**												
Polytechnic and professional	1 (Ref)											
Primary and secondary	1.16	[0.90, 1.49]	0.27	1.01	[0.94, 1.09]	0.79	1.01	[0.96, 1.06]	0.72	0.99	[0.90, 1.08]	0.77
University	0.95	[0.79, 1.13]	0.54	0.99	[0.93, 1.05]	0.72	0.99	[0.95, 1.03]	0.58	0.99	[0.92, 1.07]	0.82
**Number of children in family**	1.00	[0.90, 1.11]	0.95	1.02	[0.98, 1.05]	0.42	0.99	[0.97, 1.01]	0.47	0.97	[0.93, 1.01]	0.19
**Type of housing**												
HDB 1–3 rooms	1 (Ref)											
HDB 4–5 rooms	0.95	[0.71, 1.28]	0.76	1.01	[0.94, 1.10]	0.73	1.04	[0.98, 1.10]	0.21	1.09	[0.97, 1.23]	0.17
Private housing (condo, landlord)	1.01	[0.71, 1.44]	0.95	1.01	[0.92, 1.11]	0.84	1.09	[1.02, 1.17]	0.01	1.10	[0.96, 1.26]	0.19
**Children’s HRQOL~**							0.99	[0.99, 1.00]	p < 0.001	0.98	[0.97, 0.99]	p < 0.001

IRR: incidence rate ratio; CI: confidence interval.

*Missing data: Disease severity, n = 46; Type of housing, n = 2.

~HRQOL: disease-specific HRQOL of patients with AD was measured using IDQOL and CDLQI.

### Factors related to children’s quality of life subdomains sleep disturbance and symptoms

Table 3 presents a negative binomial analysis assessing the related sociodemographic and clinical factors in two subscales (sleep disturbance and symptoms and feelings) with the most impact on children in Fig. 2. Age of infants and long disease duration may have an impact on the subdomain of symptoms and feelings (IDQOL age: IRR: 1.03, 95% CI: [1.01, 1.06], P = 0.02; CDLQI disease duration: IRR: 1.02, 95% CI: [1.00, 1.04], P = 0.04). A greater severity of atopic dermatitis was associated with a poorer quality of life in children for symptoms’ and feelings’ subdomain (moderate cases of IDQOL: IRR: 1.24, 95% CI: [1.10, 1.41], P = 0.001; severe cases of IDQOL: IRR: 1.31, 95% CI: [1.07, 1.61], P = 0.01; moderate cases of CDLQI: IRR: 1.20, 95% CI: [1.04, 1.40], P = 0.02). Infants and children with a high severity of disease suffered from significantly more severe sleep disturbance compared to those with lesser disease severity (severe cases of IDQOL: IRR: 1.49, 95% CI: [1.19, 1.87], P = 0.001; moderate cases of CDLQI: IRR: 1.63, 95% CI: [1.25, 2.13], P<0.001; severe cases of CDLQI: IRR: 1.97, 95% CI: [1.50, 2.60], P<0.001).

**Table 3:** Multivariable regression analysis with factors associated with symptoms and feelings, and sleep disruption.

Variables	Symptoms and feelings						Sleep disturbance					
	IDQOL			CDLQI			IDQOL			CDLQI		
	IRR	95% CI	P-value	IRR	95% CI	P-value	IRR	95% CI	P-value	IRR	95% CI	P-value
**Age of child (years)**	1.03	[1.01, 1.06]	0.02	1.00	[0.97, 1,02]	0.78	0.99	[0.95, 1.03]	0.63	0.97	[0.94, 1.01]	0.13
**Gender**												
Male	1(Ref)											
Female	1.06	[0.94, 1.19]	0.35	1.11	[0.96, 1.27]	0.15	1.04	[0.89, 1.21]	0.61	1.02	[0.81, 1.28]	0.89
**Disease duration (years)**	1.01	[0.98, 1.04]	0.56	1.02	[1.00, 1.04]	0.04	0.99	[0.95, 1.04]	0.80	1.03	[1.00, 1.06]	0.05
**Severity**												
Mild	1(Ref)											
Moderate	1.24	[1.10, 1.41]	0.001	1.20	[1.04, 1.40]	0.02	1.11	[0.93, 1.33]	0.24	1.63	[1.25, 2.13]	p < 0.001
Severe	1.31	[1.07, 1.61]	0.01	1.20	[0.98, 1.47]	0.08	1.49	[1.19, 1.87]	0.001	1.97	[1.50, 2.60]	p < 0.001
**Ethnicity**												
Chinese	1(Ref)											
Indian	1.00	[0.85, 1.18]	0.97	1.07	[0.87, 1.30]	0.54	1.24	[1.02, 1.50]	0.03	1.06	[0.78, 1.45]	0.70
Malay	0.99	[0.75, 1.30]	0.93	1.08	[0.79, 1.46]	0.64	1.49	[1.18, 1.88]	0.001	1.34	[0.86, 2.07]	0.19
Others	1.12	[0.88, 1.42]	0.36	1.17	[0.87, 1.59]	0.30	1.18	[0.82, 1.69]	0.37	0.86	[0.46, 1.61]	0.63
**Educational attainment**												
Polytechnic and professional	1(Ref)											
Primary and secondary	1.04	[0.86, 1.25]	0.70	1.00	[0.85, 1.18]	0.98	1.35	[1.06, 1.72]	0.02	0.95	[0.71, 1.26]	0.72
University	0.96	[0.84, 1.11]	0.59	0.91	[0.77, 1.09]	0.32	0.97	[0.82, 1.15]	0.75	0.87	[0.65, 1.16]	0.35
**Number of children in family**	0.97	[0.90, 1.06]	0.54	0.95	[0.87, 1.03]	0.23	1.02	[0.92, 1.12]	0.76	1.04	[0.90, 1.19]	0.63
**Type of housing**												
HDB 1–3 rooms	1(Ref)											
HDB 4–5 rooms	1.07	[0.86, 1.33]	0.55	0.99	[0.82, 1.21]	0.93	0.97	[0.72, 1.30]	0.85	0.94	[0.70, 1.26]	0.67
Private housing (condo, landlord)	1.03	[0.80, 1.31]	0.84	0.78	[0.60, 1.01]	0.06	1.10	[0.77, 1.56]	0.61	0.93	[0.61, 1.42]	0.73

IRR: incidence rate ratio; CI: confidence interval.

*Missing data: Disease severity, n = 46; Type of housing, n = 2.

### Factors related to caregivers’ quality of life

**Figure 3:**
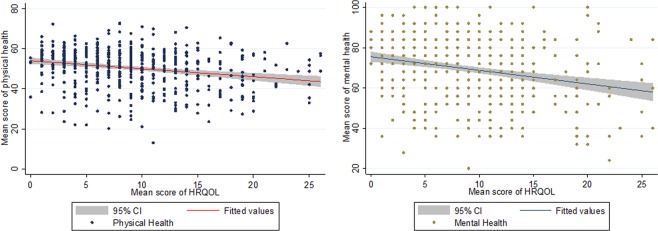
Association between children’s mean score of HRQOL and caregivers’ physical and mental health (X-axis: mean score of HRQOL; Y-axis: mean score of physical health/mental health). *HRQOL in this figure is a composite of IDQOL and CDLQI. A higher HRQOL in this figure indicates a lower quality of life.

Table 2 shows that poor health-related quality of life of children may also impair their caregivers’ mental and physical health (physical: IRR: 0.99, 95% CI: [0.99, 1.00], P < 0.001; mental: IRR: 0.98, 95% CI: [0.97, 0.99], P < 0.001). The scatterplot in Fig. 3 demonstrate the relationship between children’s HRQOL and their caregivers’ physical and mental health. It shows that as children’s HRQOL decrease, their caregivers’ mental health and physical health could drop (a higher HRQOL score here meant a lower quality of life because this score was composed combining IDQOL and CDLQI). Caregiver’s physical health might be also negatively associated with their children’s age, with an older age having a more severe effect on their physical health (IRR: 1.01, 95% CI: [1.00, 1.01], P = 0.03). Children with severe atopic dermatitis have also a significant impact on their caregiver’s physical health while mild and moderate cases did not demonstrate such an influence (IRR: 0.94, 95% CI: [0.89, 0.99], P = 0.03).

## Discussion

This study has shown that disease severity, rather than sociodemographic factors affect the health-related quality of life for children. The severity of atopic dermatitis is significantly associated with patients’ and caregivers’ self-reported quality of life: more severe atopic dermatitis is related to a poorer quality of life. Another finding emerging from this study was that caregiver’s mental and physical health was directly affected by their children’s health-related quality of life. Poor quality of life in atopic dermatitis children could lead to poor mental and physical functioning among caregivers.

A meta-analysis published in 2016 has shown the similar results with our study^17^. Besides, when it is compared to prior studies in Singapore, this study reported better quality of life (lower CDLQI/IDQOL score)^12^. The discrepancy may be because our sample size allowing us detect a more accurate effect and participants recruited from dermatology specialist clinics with more severe atopic dermatitis^18–21^. The results above also suggest that sociodemographic factors, such as caregiver’s educational attainment, may be of less influence on the treatment of children with atopic dermatitis than clinicians might intuitively think. In comparison to children, a relationship between caregiver’s physical health and type of housing was found. This trend is in line with previous studies showing that social status may affect adults more than their children^22,23^. In research exploring the relationship between childhood atopic dermatitis and its impact on parents health similar conclusions have been described: a strong relationship was found between a greater disruption of sleep of the child with atopic dermatitis and higher levels of maternal anxiety and depression^8^. Due to the burden of the condition and the impact of new routines imposed by continuous treatment, caregivers have to restructure their lives^24^. Parents may spend hours to comfort their child and manage the disease. Adaptation to these changes also requires parents to be prepared mentally and physically for sleep disturbance, emotional distress, and exhaustion^25,26^. It is worrying that the negative impact on quality of life in these caregivers is more severe or comparable to prevalent paediatric chronic diseases, such as psychiatric disease and congenital heart disease^8,15,16^. Our results indicate the traditional scope in the treatment of atopic dermatitis should be broadened to include not only affected children but also their surrounding support system.

This in-depth study is the first to assess quality of life subdomains and related clinical and sociodemographic factors. Itching/scratching, emotional distress, and sleep disturbance were quality of life subdomains most reported, and their reporting increased with higher disease severity (Fig. 2). Besides, our study also showed that greater severity of atopic dermatitis leads to poorer health-related quality of life of the child. This might be explained by the fact that affected subdomains could be considered as a cluster issue. Symptoms such as intractable itching and scratching can consequently aggravate atopic dermatitis lesions resulting in significant sleep deprivation leading to exhaustion, unstable emotions and impaired functioning^3,27^. However, caregivers’ quality of life is affected by multiple factors and could be directly affected by their children’s quality of life instead of disease severity. As such, treatment strategies targeting specific atopic dermatitis symptoms should improve patient and caregiver quality of life.

Findings from this study have shown that the management and treatment routine of atopic dermatitis needs to be adapted to incorporate quality of life assessment in affected patients. In addition, preventing deterioration of the disease seems a key point in treatment. However, complete severity and symptom assessment and monitoring are not generally adopted in clinical routine, as this process is seen as too time-consuming. Disease tracking is an important factor in the treatment of atopic dermatitis, but self- or carers- assessment may be unreliable without appropriate guidance. Therefore, more convenient and efficient tools need to be deployed in the future to meet the current needs. Moreover, treatment should focus beyond the disease and more attention should be directed to the symptoms and feelings of children and their caregivers. Effective, evidence-based psychosocial assessment and intervention tools should be made available. For example, health apps could be tailored to target user’s tracking needs as the diagnosis and management of atopic dermatitis are mainly based on visual examination^28^. An app could also be valuable for keeping patients engaged, educate them and create awareness of their skin status. It would provide an opportunity for healthcare professionals to monitor and conduct (symptoms and feelings) interventions and guidance in real-time on a distance^29,30^.

It is interesting for us to find that moderate AD significantly decreased QOL while no similar results were observed in severe cases in CDLQI score. This finding appeared unexpected as clinical observations would suggest that individuals with more severe symptoms should be affected more than those with less severity. This could be explained: (1) Doctors subscribe stronger medications to treat the symptoms such as itchiness and sleep problems and therefore children with severe disease might be less affected by AD. (2) Severe patients and their caregivers may have better adherence to therapy and hence report to be less affected by severe AD. (3) More severe individuals might be get used to the poor quality of life as well as have lower expectations than those with mild to moderate symptoms, and this would lead them to report better quality of life. Similar trends have been reported in the relation of quality of life to sleep and obsessive-compulsive disorder and other mental health issues^31,32^.

This study has several strengths. The high recruitment rate and the large study sample provide a good representation of children with atopic dermatitis in Singapore. According to our power calculation, the sample size of 559 participants is sufficient to capture the quality of life status accurately to allow subgroup analysis and to be generalised to other high prevalence countries. To our knowledge, this is one of the largest atopic dermatitis quality of life studies performed in both children with atopic dermatitis and their caregivers. Secondly, this study is the first to employ a negative binomial model to test causation between social, clinical factors and paediatric atopic dermatitis patients’ HRQOL. This model highlights how paediatric atopic dermatitis patients’ health-related quality of life affects their caregivers’ quality of life, which leads to a more complete understanding of the total burden. A weakness of this study is the lack of a non-atopic dermatitis control group. Therefore, we cannot compare atopic dermatitis patients and caregivers HRQOL with non-atopic dermatitis children and caregivers. We have however attempted to compare caregiver’s quality of life for other chronic diseases. Thirdly, a cross-sectional study may preclude concrete observation regarding the influence of atopic dermatitis over time. Lastly, the IDQOL scores may not accurately detect infants’ HRQOL as their caregivers filled in the questionnaire on behalf of their infants.

## Conclusion

This study provides an in-depth view of the atopic dermatitis-related quality of life among children and their caregivers in Singapore. It helps us understand the complex relationship between sociodemographic factors, disease severity, and quality of life in atopic dermatitis patients. More attention should be directed to atopic dermatitis symptoms and their effect on daily life. The correlation between disease severity and quality of life implicates that treatment should focus on prevention of severe atopic dermatitis. Future studies should investigate interventions to address factors influencing both the quality of life and the disease severity of children and their caregivers.

## Methods

### Design and participants

A cross-sectional survey was conducted from December 2016 to December 2017 at two paediatric dermatology clinics in Singapore: KK Women’s and Children’s Hospital and National Skin Centre. Children and their caregivers were invited to participate in this study. Patients were eligible if they were (i) 0 to 16 years of age and (ii) fulfilling the Hanifin and Raijka criteria (1980) for atopic dermatitis^33^, (iii) able to understand English/Mandarin (Singapore has several official languages with majority of population speaking English or Chinese), (iv) had approved consent by their caregiver. Caregivers were included if they were: (i) equal to or older than 21 years of age, (ii) able to understand English/Mandarin, (iii) the legal guardian.

### Instruments

The following information and instruments were included in the study: 36-item short form survey (RAND-36) being one of the most widely used health-related quality of life (HRQOL) instruments and has been validated for Singapore population^34,35^. The Infants’ Dermatitis Quality of Life Index (IDQOL) measures health-related quality of life of children below the age of four. IDQOL has been validated in a Chinese population in China, but it has not yet been validated in Singapore^36^. For children above four years of age, who were not able to understand the questionnaires, their caregivers were asked to fill in IDQOL on behalf of the children. The Children’s Dermatology Life Quality Index (CDLQI) is a widely used questionnaire to measure the quality of life of children aged from four to 16 years. CDLQI has been validated in Mandarin in Hongkong, but it has not yet been validated in Singapore^37^. Details of each scoring system can be found in Table 4.

**Table 4:** Study instruments and scoring system.

● 36-item short form survey (RAND-36)
The score ranges from 0 to 100, with a lower score indicating poorer health or functioning^41^.
● The Infants’ Dermatitis Quality of Life Index (IDQOL)
The questionnaire contains ten questions covering six areas of daily activities including symptoms and feelings, leisure, school or holidays, personal relationships, sleep, and treatment^36^. Each question is answered on a 4-point Likert scale scored from 0 to 3. These are added to give a minimum score of 0 and a maximum score of 30. A higher score indicates a greater degree of QoL impairment^36^. The severity banding for IDQOL scores: 0–1 = no effect on infant’s life; 2–5 = small effect; 6–10 = moderate effect; 11–20 = very large effect; 21–30 = extremely large effect^42^.
● The Children’s Dermatology Life Quality Index (CDLQI)
The questionnaire contains ten questions covering six areas of daily activities including symptoms and feelings, leisure, school or holidays, personal relationships, sleep, and treatment^1^. Each question is answered on a 4-point Likert scale scored from 0 to 3. These are added to give a minimum score of 0 and a maximum score of 30. A higher score indicates a greater degree of QoL impairment^1^. The severity banding for CDLQI scores: 0–1 = no effect on child’s life; 2–6 = small effect; 7–12 = moderate effect; 13–18 = very large effect; 19–30 = extremely large effect^1,43^.

### Disease severity assessment

Eczema severity was extracted from the electronic medical records. Information from medical records was extracted at the same time point as caregivers assessed their child’s current condition of AD. At both study settings, physicians applied a modified physician global assessment (PGA) when rating the severity of AD: Clear refers to ‘no inflammatory signs of AD’; Almost clear refers to ‘faint, barely detectable erythema and/or trace residual induration/papulation in limited areas; neither excoriation nor oozing/crusting are present’; Mild refers to ‘light pink erythema and slightly perceptible induration/papulation; excoriation are present’; Moderate refers to ‘dull red, clearly distinguishable erythema and clearly perceptible induration/papulation but not extensive; excoriation or oozing/crusting are present’; Severe refers to ‘deep/dark red erythema, and marked and extensive induration/papulation; excoriation and oozing/crusting are present’. For those patients whose severity was not explicitly reported in the electronic medical records, symptoms and affected area were extracted from electronic medical records and assessed by an investigating physician researcher (XX) using Eczema Area and Severity Index (EASI) calculator into mild (EASI score 1.1–7.0), moderate (EASI score 7.1–21.0) and severe (EASI score 21.1–50.0), very severe (EASI score 50.1–72)^38,39^. This score is calculated by the percentage of skin affected by eczema for each body region and the intensity scores of four signs: redness; thickness; scratching; lichenification^38^.

### Statistical analysis

According to the sample size formula for a qualitative variable^13,40^, a sample size of at least 518 children could effectively detect and quantify relevant parameters, and allow for subgroup analyses. Descriptive statistics for continuous variables are reported with mean ± SD, categorical variables are reported with frequencies and percentages (%). Normality of the continuous variables was tested by the Shapiro–Wilk test. Wilcoxon rank-sum (or Mann–Whitney) test for two groups and Kruskal-Wallis test for more than two groups were used to find statistical association between sociodemographic characteristics (ethnicity, education, occupation, housing accommodation) and RAND-36, CDLQI and IDQOL score along with their subdomains. Subsequently, a negative binomial regression model was used to demonstrate the relationship between sociodemographic variables and CDLQI, IDQOL and RAND-36 measures. Univariable and multivariable incidence rate ratios (IRR) were calculated and reported with 95% confidence intervals (CIs). Statistical analysis was carried out using Stata 14.2 (StataCorp, College Station, TX, USA). Two-sided *p*-value less than 0.05 was taken as statistically significant and the 95% confidence intervals (CIs) are presented.

### Ethical approval and informed consent

The present study was approved by the Institutional Review Board of National Healthcare Group (NHG-DSRB: 2015/01228) and Nanyang Technological University (NTU IRB: IRB-2016-10-059-01). The methods were carried out in accordance with the relevant guidelines and regulations. Informed consent was obtained from all participants and their legal guardians. identifiers must be removed from all sections of the manuscript, including Supplementary Information. In order to protect the anonymity of their responses, no IP addresses, email addresses, or identifying information was collected.

## Supplementary information

STROBE checklist

## Data Availability

All data and materials are publicly accessible.
